# Age-Related Frontal Periventricular White Matter Hyperintensities and miR-92a-3p Are Associated with Early-Onset Post-Stroke Depression

**DOI:** 10.3389/fnagi.2017.00328

**Published:** 2017-10-05

**Authors:** Ji-Rong He, Yu Zhang, Wen-Jing Lu, Huai-Bin Liang, Xuan-Qiang Tu, Fei-Yue Ma, Guo-Yuan Yang, Li-Li Zeng

**Affiliations:** ^1^Department of Neurology, Ruijin Hospital, Shanghai Jiao Tong University School of Medicine, Shanghai, China; ^2^Department of Neurology, Ruijin Hospital Luwan Branch, Shanghai Jiao Tong University School of Medicine, Shanghai, China

**Keywords:** depression, microRNA, stroke, white matter hyperintensities, frontal caps

## Abstract

**Objective:** To explore the correlationship among white matter hyperintensities (WMHs), miR-92a-3p and early-onset post-stroke depression (PSD).

**Methods:** We recruited consecutively 238 patients with acute cerebral infarction and MRI examination in the Department of neurology, Ruijin hospital, Shanghai Jiaotong University School of Medicine. The diagnosis of early-onset PSD was made in accordance with DSM-IV criteria for depression in 2 weeks after stroke. Clinical information and assessments of stroke severity were recorded on admission. The analysis of plasma miR-92a-3p was performed using quantitative PCR at the same time. WMHs were evaluated by the Fazekas and Scheltens visual rating scales. The relationship among WMHs, miR-92a-3p and PSD were analyzed by SPSS 22.0 software.

**Results:** Logistic regression demonstrated that periventricular WMHs (PVWMHs) in frontal caps was an independent risk factor with early-onset PSD (OR = 1.579, 95% CI: 1.040–2.397, *p* = 0.032). The age and numbers of lacunes were related to frontal PVWMHs. Plasma miR-92a-3p in the PSD group was higher compared with the non-depressed group. Receiver operating curve analysis revealed that miR-92a-3p could predict early-onset PSD with 90% sensitivity and 90% specificity. The higher miR-92a-3p trended toward association with greater frontal PVWMHs.

**Conclusion:** Acute ischemic stroke patients with frontal PVWMHs or a high plasma miR-92a-3p at baseline were more likely to develop early-onset PSD. MiR-92a-3p might be involved in the white matter impairment and post-stroke depression.

## Introduction

As a common complication of stroke, post-stroke depression (PSD), characterized by long-lasting persistent low mood, is an affective disorder associated with stroke events. The morbidity of PSD ranges from 25 to 79% (Esparrago Llorca et al., 2015; Robinson and Jorge, 2016). PSD could occur from several weeks to years following ischemia. Early-onset PSD refers to suffering from depression within 2 weeks after stroke onset. Delaying of recovery of neurological functions (Herrmann et al., 1998; Perez and Tardito, 2002; Turner-Stokes and Hassan, 2002), impact on activities of daily living and quality of life (Parikh et al., 1990; Chemerinski et al., 2001), the growth of stroke recurrence (Larson et al., 2001; May et al., 2002) and mortality (Morris et al., 1993; House et al., 2001; Williams et al., 2004), can be found in PSD patients. However, the pathogenesis of PSD remains unclear. Studies revealed that the amine hypothesis, neurotransmitter and neurotrophin signaling, hippocampal neurogenesis, cellular plasticity in the ischemic lesion, secondary degenerative changes, activation of the HPA axis and neuroinflammation might be involved in PSD (Loubinoux et al., 2012). Therefore, it is important to elucidate the pathogenesis, identify the related risk factors and explore novel biomarkers for the diagnosis and treatment of PSD.

As one form of cerebral small vessel disease (CSVD), white matter hyperintensities (WMHs), also known as white matter lesions (WML) or leukoaraiosis (LA), refer to the punctate or patchy changes mainly in periventricular and subcortical white matter of the brain, and could be observed on MRI-T2 weighted and fluid attenuated inversion recovery (FLAIR) images as hyperintense lesions. The presence and severity of WMHs were associated with many underlying microstructural processes, such as chronic ischemia resulted from venous collagen deposition and impaired cerebral blood flow autoregulation, blood–brain barrier damage, neural inflammation and immunity, and it was an extreme consequence of such processes that affected brain connectivity (Lin et al., 2017). WMHs are closely related to cognitive impairment, gait abnormalities and urinary incontinence (Zheng et al., 2011; Lin et al., 2017). There are several reports on the correlation between WMHs and PSD, however, the conclusions are inconsistent (Vataja et al., 2001; Nys et al., 2005; Tang et al., 2010). This may be related to different study design, time point of assessment, evaluation methods or research subjects. The assessment time point could be 2 weeks, 3 months, 1 year or even several years. There were also differences in the evaluation methods for depression and WMHs. The subjects came from different types of hospitals such as rehabilitation hospitals, stroke centers, community hospitals and general hospitals. The illness severity and the type of stroke varied, too. Furthermore, the small sample size which undermined generalization of the main findings, the lack of accounting for the cognitive effects of medications, the presence of methodological shortcomings such as the low spatial resolution of the imaging equipment, etc. All the limited conditions above could lead to inconsistent results.

MicroRNAs (miRNAs), with a length of about 22 nucleotides, are small non-coding RNAs that bind to specific mRNAs to regulate gene expression in various organisms. Studies showed that miRNAs were key players involved in nervous system development, physiology, and disease. It played a critical role in major depression and suicidal behavior which is frequently related to major depression as a negative outcome. The circulating miRNA was a novel potential blood biomarker in many diseases including major depression and suicidal behavior (Serafini et al., 2014; Schulte and Zeller, 2015; Witwer, 2015). Serafini et al. (2014) summarized that the reduced expression of miRNAs was found in the prefrontal cortex of depressed suicide patients. The differently expressed miRNAs influence the homeostasis of neural and synaptic pathways by negatively regulating gene expression such as CREB-BDNF pathways and the other important signaling pathways (PKC, PTEN, ERK-MAP kinase, Wnt/b-catenin etc.). MiRNAs downregulated the expression of VEGFA signaling proteins, ion channels, ubiquitin ligases and transcription factors such as NOVA1, critically involved in neurotransmitter release, synaptic plasticity and major depression (Serafini et al., 2014). Our previous study of microRNA expression profile firstly demonstrated that miR-92a-3p was one of the 25 differential expressed blood miRNAs in early-onset PSD patients, compared with the non-depressed group (Zhang et al., 2016). But the diagnostic efficacy and possible mechanisms remain unknown. MiR-92a-3p has been reported to be involved in pathophysiological processes such as endothelial dysfunction, lipid metabolism and atherosclerosis (Daniel et al., 2014; Loyer et al., 2014). WMHs were closely related to hypertension. Researches revealed that endothelial dysfunction, vascular stiffness, blood-brain barrier abnormalities, and glia damage were involved in the pathophysiology of WMHs (Rincon and Wright, 2014). Here, we hypothesized that cerebral white matter impairment might be one target of miR-92a-3p leading to PSD.

In view of the pathophysiological basis and previous studies of PSD, WMHs and miR-92a-3p mentioned above, for the aim of the present study, we wanted to clarify that patients with WMLs or elevated miR-92a-3p at baseline were more likely to develop depression within 2 weeks after stroke, and white matter impairment was possibly to be one of the mechanisms of miR-92a-3p in the pathogenesis of PSD. We also tried to confirm whether miR-92a-3p could serve as a novel biomarker and a potential therapeutic target of PSD.

## Materials and Methods

### Study Subjects

The study was conducted in 238 acute cerebral infarction patients with magnetic resonance imaging scans performed in the Department of neurology, Ruijin hospital, Shanghai Jiaotong University School of Medicine from May 2013 to September 2014, including 162 males and 76 females, aged 32 to 91 years. Inclusion criteria: (1) The diagnosis of ischemic stroke conformed to the guideline for the diagnosis and treatment of acute ischemic stroke in China in 2010, and was confirmed by head MRI scans. (2) The course of disease was less than 1 week. (3) Over 18 years of age. Exclusion criteria: (1) Patients accompanied by unconsciousness, aphasia or severe cognitive impairment could not cooperate with the examination. (2) Patients with psychosis or other psychiatric conditions such as anxiety, depression and suicidal behavior. (3) Patients with other severe systemic diseases including infection, cardiac and pulmonary failure, or hepatic and renal dysfunction. (4) Patients failed to perform MRI scans for various reasons. The study protocol was approved by the Ethics Committee of Ruijin Hospital, Shanghai Jiaotong University School of Medicine. All participants signed a written consent form. The Hamilton depression-17 scale (HAMD-17) and the clinical interview were performed in 2 weeks after stroke onset by a trained neurologist. HAMD-17 score ≥ 7 was considered to be depressive. The diagnosis of PSD was in accordance with the Diagnostic and Statistical Manual IV (DSM-IV) criteria for depression.

### Methods

#### Clinical Data Collection

The baseline clinical data of the subjects was enrolled, including age, gender, education, hypertension, diabetes, hyperlipidemia, heart disease, location of stroke, size of acute infarction and Trial of Org 10 172 in acute stroke treatment (TOAST) classification on the day of admission. The National Institutes of Health Stroke Scale (NIHSS) was scored synchronously. On the day after admission, fasting vein blood 4 ml was collected, and blood laboratory examinations, including white blood cell (WBC) count, fasting blood glucose, glycosylated hemoglobin (HbA1c), total cholesterol (TC), triglyceride (TG), low density lipoprotein cholesterol (LDL-C), high density lipoprotein cholesterol (HDL-C), apolipoprotein A (ApoA), and apolipoprotein B (ApoB), were completed. The HAMD-17 scale was used to assess depressive symptoms 2 weeks after onset. TOAST classification was reconfirmed at the time of discharge.

Diagnostic criteria for risk factors of stroke: (1) Hypertension: The blood pressure was measured at the upper arm and brachial artery, and elevated at least two times (systolic pressure > 140 mmHg and/or diastolic pressure > 90 mmHg) by three times repeated measurements on different days. Or there was a definite history of hypertension, and medication was used to keep blood pressure within normal limits. (2) Diabetes: (a) There were symptoms of diabetes, and the venous plasma glucose concentration ≥11.1 mmol/L at any time; (b) The fasting venous plasma glucose concentration ≥7.0 mmol/L; (c) The venous plasma glucose concentration ≥11.1 mmol/L 2 h after the execution of oral glucose tolerance test (OGTT). Of the above three criterions, one or more of them met the standards. Besides, the criteria should be met again by repeat examination of one or more of the above three items on the following day. Or there was a definite history of diabetes, and medication was used to keep blood glucose within normal limits. (3) Hyperlipidemia: TG >1.7 mmol/L (0.56–1.7 mmol/L) and/or TC >5.7 mmol/L (2.33–5.7 mmol/L) and/or LDL-C >4.3 mmol/L (1.3–4.3 mmol/L). Or there was a definite history of hyperlipidemia, and medication was used to keep blood lipid within normal limits. (4) Heart disease: There was a definite history of heart disease, including coronary heart disease, valvular disease and arrhythmia. Or no definite history but objective evidences on clinical abnormalities, such as electrocardiogram, echocardiography, etc.

TOAST classification of ischemic stroke: There were 5 etiological types of ischemic stroke: (1) Large artery atherosclerosis (LAA): Intracranial and extracranial aortic stenosis ≥50%, infarct diameter ≥15 mm. (2) Cardiac embolism (CE): Cerebral embolism resulting from a variety of heart diseases that could produce cardiogenic emboli. (3) Small artery occlusion (SAO): Ischemic stroke caused by stenosis or occlusion of intracranial arteriole, infarct diameter <15 mm. (4) Stroke of other determined etiology (SOD): Ischemic stroke caused by infection, immunity, non-immune vascular disease, hypercoagulability, hematologic diseases, hereditary vascular diseases and drug taking. (5) Stroke of undetermined etiology (SUD): Multiple examinations failed to reveal the cause of the disease.

#### Assessment of WMHs

1.5-T Head MRI scans were performed within 3 days of admission. WMHs were assessed by means of Fazekas and Scheltens visual rating scales by one qualified neurologist via MR images.

According to the methods of Fazekas et al. (1987), WMHs were divided into periventricular white matter hyperintensities (PVWMHs) and deep white matter hyperintensities (DWMHs). PVWMHs scoring criteria: 0 point: no WMHs; 1 point: caps or pencil-thin lining; 2 points: smooth halo; 3 points: irregular PVWMHs extending into the deep white matter. DWMHs scoring criteria: 0 point: no WMHs; 1 point: punctate foci; 2 points: beginning confluence of foci; 3 points: large confluent areas. Total score: PVWMHs score + DWMHs score.

According to the methods of Scheltens et al. (1993), WMHs were divided into four areas: PVWMHs, DWMHs, basal ganglia WMHs and infratentorial WMHs. (1) PVWMHs scoring criteria (0–6): occipital caps (0–2); frontal caps (0–2); bands (0–2). 0 point: no WMHs; 1 point: lesion <6 mm; 2 points: lesion 6–10 mm; lesions over 10 mm were recorded as DWMHs. Three items added up to get the total PVWMHs score. (2) DWMHs scoring criteria (0–24): frontal (0–6); parietal (0–6); occipital (0–6); temporal (0–6). 0 point: no WMHs; 1 point: lesion <4 mm, *n* ≤ 5; 2 points: <4 mm, *n* > 5; 3 points: 4–10 mm, *n* ≤ 5; 4 points: 4–10 mm, *n* > 5; 5 points: >10 mm, *n* ≥ 1; 6 points: large confluent lesions. Four items added up to get the total DWMHs score. (3) Basal ganglia WMHs scoring criteria (0–30): caudate nucleus (0–6); putamen (0–6); globus pallidus (0–6); thalamus (0–6); internal/external capsule (0–6). Assessment methods ibid. (4) Infratentorial WMHs scoring criteria (0–24): cerebellum (0–6); mesencephalon (0–6); pons (0–6); medulla (0–6). Assessment methods ibid. (5) Total score: PVWMHs score + DWMHs score + basal ganglia WMHs score + infratentorial WMHs score.

In addition, the number of old lacunar infarctions in the brain was counted by the head MRI. And ventricle-to-brain ratio was calculated (de Groot et al., 2000). Ventricle-to-brain ratio = [(width of anterior horns of lateral ventricle/corresponding brain width at the same level) + (biventricular width at the level of the body of caudate nucleus/corresponding brain width at the same level) + (width of occipital horns of lateral ventricle/corresponding brain width at the same level)]/3.

#### Detection of microRNAs

In the early-onset post-stroke depression group and non-depression group, 20 patients per group (40 in all) were selected to take plasma miR-92a-3p detected. There was no significant difference in gender, age, stroke location, NIHSS score and TOAST type between the two groups (all the *p*-values >0.05).

Four milliliter venous blood of the patients was collected into an EDTA anticoagulant tube on the morning of the day after admission. The blood sample was centrifuged by 3000 rpm immediately. The upper plasma was packed with an EP tube 15 min later and was stored in a -80°C refrigerator. Total RNA was isolated with mirVanaTM RNA Isolation Kit (Applied Biosystem p/n AM1556, United States) in accordance with the operating manual of the kit. The synthesis of cDNA was carried out in the reverse transcription system in the 0.2 ml PCR tube (Axygen PCR-02-C, United States). The reaction system was as follows: total RNA 0.5 μg, 5×miScript HiSpec Buffer 2 μl, 10×Nucleics Mix 1 μl, miScript Reverse Transcriptase Mix 0.5 μl, Nuclease-free H_2_O up to 10 μl, 37°C 60 min reverse transcription on the PCR machine (ABI 9700, United States), 95°C 5 min terminated the reaction. After reverse transcription, diluted cDNA with nuclease free water at 1:10: 1 μl cDNA+ 9 μl nuclease free water. Mixed the reagents: 2×LightCycler 480 SYBR Green I Master (Roche, Swiss) 5 μl, 10 μM Universal primer (Qiagen, Germany) 0.2 μl, 10 μM microRNA-specific primer (Generay, China) 0.2 μl, cDNA 1 μl, Nuclease-free H_2_O 3.6 μl, total volume 10 μl. Added ROX reference dye 0.2 μl. Inserted the sample on the 384 hole plate and seal the film, centrifuged it for 1 min at 1500 *g*, removed bubbles, then performed the RT-PCR amplification. Cycling program: thermal cycler (Roche LC480II, Swiss); pre-incubation: 95°C 10 min; denaturation, annealing and extension: 95°C 10 s, 60°C 30 s, each of 40 cycles. The fluorescence quantitative PCR machine ran on its own software to analyze the melting curve. At the end of the reaction, the PCR reaction curve was analyzed and the *C*t-value was obtained. Using cel-miR39 as internal reference, calculation of the target gene expression was conducted by 2^-ΔΔCt^ relative quantification method.

### Data Analysis

SPSS 22.0 statistical software was adopted to process and analyze the data. The normality was checked by Kolmogorov–Smirnov test. Measurement data of normal distribution, expressed as mean ± standard deviation (-x ± s), was checked by two independent samples *t*-test. Measurement data of non-normal distribution, expressed as median and interquartile range [*M*(P25,P75)], was checked by rank-sum test. Count data was expressed as constituent ratio (%) or ratio (%). The correlation between WMHs and PSD was analyzed by multiple logistic regression analysis. The receiver operating curve (ROC) was drawn to analyze the diagnostic efficacy of miR-92a-3p. The level of significance was set at 0.05.

## Results

### General Data of Patients

Two hundred and thirty-eight patients were enrolled, including 162 males and 76 females, aged 32 ∼ 91 years, with an average of 66 ± 11 years. Of the 238 patients, 42 suffered from depression 2 weeks after stroke, while the other 196 did not, and the morbidity of early-onset PSD was 17.6%. PSD group (*n* = 42) and non-depressed group (*n* = 196) did not differ in age, gender, education, hypertension, diabetes, hyperlipidemia, heart disease, location of stroke, lateralization, size of acute infarction and TOAST classification (*p* > 0.05, **Table 1**), while there was significant difference in NIHSS score (NIHSS > 3) between the two groups (*p* = 0.000, **Table 1**).

**Table 1:** Clinical data comparisons between post-stroke depression (PSD) group and non-depressed group.

Variable	PSD group *n* = 42	Non-depressed group *n* = 196	*P*-value
**Demography**			
Age (years)	67.57 ± 10.36	66.54 ± 11.16	0.222
Gender (Male/Female)	26/16	136/60	0.346
Education (High school and above) (%)	23 (54.8)	109 (55.6)	0.920
**Baseline vascular risk factors**			
Hypertension (%)	36 (85.7)	145 (74.0)	0.107
Diabetes mellitus (%)	14 (33.3)	64 (32.7)	0.932
Hyperlipidemia (%)	20 (47.6)	93 (47.5)	0.984
Heart diseases (%)	7 (16.7)	28 (14.3)	0.693
**Stroke characteristics**			
Acute infarct (%)			0.236
Small infarct (<15 mm)	20 (47.6)	113 (57.7)	
Large infarct (≥15 mm)	22 (52.4)	83 (42.3)	
Location of acute infarct (%)			0.376
Cortical	9 (21.4)	45 (23.0)	
Subcortical white matter	4 (9.5)	22 (11.2)	
Deep	16 (38.1)	86 (43.9)	
Infratentorial	13 (31.0)	43 (21.9)	
Lateralization (%)			0.070
Dominant hemisphere	17 (40.5)	102 (52.0)	
Non-dominant hemisphere	20 (47.6)	89 (45.4)	
Bilateral hemisphere	5 (11.9)	5 (2.6)	
TOAST subtype (%)			0.547
LAA	20 (47.6)	101 (51.5)	
CE	0 (0.0)	9 (4.6)	
SAO	15 (35.7)	55 (28.1)	
SOD	0 (0.0)	1 (0.5)	
SUD	7 (16.7)	30 (15.3)	
NIHSS > 3 (%)	28 (66.7)	68 (34.7)	0.000

*LAA, large artery atherosclerosis; CE, cardiac embolism; SAO, small artery occlusion; SOD, stroke of other determined etiology; SUD, stroke of undetermined etiology; NIHSS = National Institutes of Health Stroke Scale*.

### Scheltens Frontal PVWMH Scores Were Associated with PSD

Between PSD group and non-PSD group, there was no significant difference in Fazekas PVWMHs score, Fazekas DWMHs score, Fazekas total score, Scheltens PVWMHs total score, occipital caps score, bands score, Scheltens DWMHs total score, frontal score, parietal score, occipital score, temporal score, Scheltens basal ganglia WMHs score, Scheltens infratentorial WMHs score, Scheltens total score, number of lacunes and Ventricle-to-brain ratio (*p* > 0.05, **Table 2**), whereas the Scheltens score of frontal PVWMHs in the PSD group was higher than that in the non-depressed group (*p* = 0.044, **Table 2**).

**Table 2:** Comparisons of white matter hyperintensities (WMHs), number of lacunes and ventricle-to-brain ratio between PSD group and non-depressed group.

Variable	PSD group *n* = 42	Non-depressed group *n* = 196	*p*-Value
**WMHs score according to Fazekas**			
Fazekas PVWMHs total score	1 (1,1)	1 (0,1)	0.290
Fazekas DWMHs total score	1 (1,2)	1 (1,1)	0.239
Fazekas total score	2 (2,3)	2 (1,3)	0.259
**WMHs score according to Scheltens**			
Scheltens PVWMHs total score	3 (1,4)	2 (0,4)	0.295
Occipital caps	0.5 (0,2)	0 (0,2)	0.825
Frontal caps	2 (1,2)	1 (0,2)	0.044
Bands	0 (0,0)	0 (0,0)	0.732
Scheltens DWMHs total score	5 (1,8)	4 (1,7)	0.292
Frontal	2 (1,4)	2 (1,3)	0.446
Parietal	1 (0,2.25)	1 (0,2)	0.161
Occipital	0 (0,1)	0 (0,1)	0.856
Temporal	0 (0,1)	0 (0,1)	0.626
Scheltens basal ganglia WMHs	0 (0,0)	0 (0,0)	0.710
Scheltens infratentorial WMHs	0 (0,0)	0 (0,0)	0.421
Scheltens total score	7 (3,11.25)	6 (2,11)	0.274
**Number of lacunes**	1 (0,2)	1 (0,2)	0.574
**Ventricle-to-brain ratio**	0.20 (0.18,0.22)	0.20 (0.19,0.22)	0.689

*WMHs, white matter hyperintensities; PVWMHs, periventricular white matter hyperintensities; DWMHs, deep white matter hyperintensities*.

The Scheltens score of periventricular frontal caps was further used as an independent variable for the Logistic regression model. And logistic regression analysis showed that it had a close correlation with early-onset PSD (OR = 1.518, 95% CI: 1.013–2.273, *p* = 0.043, **Table 3**). Considering the significant difference in NIHSS score (NIHSS > 3) between the two groups, we incorporated NIHSS score into the logistic regression model as an independent variable to conduct adjusted logistic regression analysis. Adjusted logistic regression analysis demonstrated that the PVWMH in frontal caps was a significant independent predictor for early-onset PSD (adjusted OR = 1.579, 95% CI: 1.040-2.397, *p* = 0.032, **Table 3**).

**Table 3:** Logistic regression analysis on related factors of early-onset PSD.

Variable	*B*-value	*SE* value	Wald value	*p*-value	OR	CI (95%)
Scheltens score of frontal caps	0.417	0.206	4.098	0.043	1.518	1.013–2.273
Constant	-2.083	0.337	38.278	0.000		
NIHSS > 3	1.367	0.365	14.022	0.000	3.922	1.918–8.020
Scheltens score of frontal caps	0.457	0.213	4.600	0.032	1.579	1.040–2.397
Constant	-2.828	0.424	44.545	0.000		

### The Age and Numbers of Lacunes Were Related with Frontal PVWMHs

According to the results of the Scheltens score of periventricular frontal caps, the total 238 recruited subjects were divided into two groups. A group of patients with a score greater than or equal to 1 point was defined as WMH group (*n* = 160), the other group (score = 0) was defined as non-WMH group (*n* = 78). Between WMH group and non-WMH group, there was no significant difference in gender, education, WBC, fasting blood glucose, HbA1C, TC, HDL-C, LDL-C, ApoA, and ApoB (*p* > 0.05, **Table 4**), while there was significant difference in age, hypertension, diabetes mellitus, hyperlipidemia, NIHSS score (NIHSS > 3), previous history of stroke, number of lacunes, ventricle-to-brain ratio and TG (*p* < 0.05, **Table 4**).

**Table 4:** Related factors of PVWMHs in frontal caps.

Variable	WMH group *n* = 160	Non-WMH group *n* = 78	*p*-value
Age (years)	69.19 ± 9.72	59.13 ± 10.52	0.000
Gender (Male/Female)	105/55	57/21	0.248
Education (High school and above) (%)	83 (51.9)	49 (62.8)	0.112
Hypertension (%)	128 (80.0)	53 (67.9)	0.041
Diabetes mellitus (%)	60 (37.5)	18 (23.1)	0.026
Hyperlipidemia (%)	67 (41.9)	46 (59.0)	0.013
NIHSS > 3 (%)	64 (40.0)	32 (41.0)	0.000
Previous history of stroke (times)	0 (0,0)	0 (0,0)	0.045
Number of lacunes	1 (0,2)	0 (0,1)	0.000
Ventricle-to-brain ratio	0.21 (0.19,0.22)	0.20 (0.18,0.21)	0.000
WBC (×10E9/L)	6.87 ± 1.84	7.24 ± 2.12	1.191
Fasting blood glucose (mmol/L)	5.3 (4.7,6.7)	5.2 (4.8,6.3)	0.366
HbA1C (%)	6.0 (5.6,7.1)	5.9 (5.5,7.3)	0.476
TG (mmol/L)	1.76 ± 0.85	2.09 ± 1.23	0.045
TC (mmol/L)	4.77 ± 1.08	4.82 ± 1.08	0.843
HDL-C (mmol/L)	1.10 ± 0.28	1.06 ± 0.30	0.097
LDL-C (mmol/L)	2.98 ± 0.92	3.04 ± 0.94	0.827
ApoA (g/L)	1.19 ± 0.21	1.18 ± 0.20	0.996
ApoB (g/L)	1.01 ± 0.26	1.04 ± 0.26	0.430

*WBC, white blood cell; HbA1c, glycosylated hemoglobin; TG, triglyceride; TC, total cholesterol; HDL-C, high density lipoprotein cholesterol; LDL-C, low density lipoprotein cholesterol; ApoA, apolipoprotein A; ApoB, apolipoprotein B*.

Further logistic regression analysis on risk factors of PVWMHs in frontal caps was conducted. We incorporated age, hypertension, diabetes mellitus, hyperlipidemia, NIHSS score (NIHSS > 3), previous history of stroke, number of lacunes, ventricle-to-brain ratio and TG into the logistic regression model as independent variables. The results demonstrated that age and numbers of lacunes were two independent risk factors of frontal PVWMHs (OR = 1.100, 95% CI: 1.054–1.148, *p* = 0.000, and OR = 1.712, 95% CI: 1.247–2.352, *p* = 0.001, respectively, **Table 5**).

**Table 5:** Logistic regression analysis on risk factors of PVWMHs in frontal caps.

Variable	*B*-value	*SE* value	Wald value	*p*-value	OR	CI (95%)
Age	0.096	0.022	19.305	0.000	1.100	1.054–1.148
Number of lacunes	0.538	0.162	11.039	0.001	1.712	1.247–2.352
Constant	-5.531	1.851	8.930	0.003		

### Plasma miR-92a-3p at Baseline Could Predict Early-Onset PSD

**FIGURE 1:**
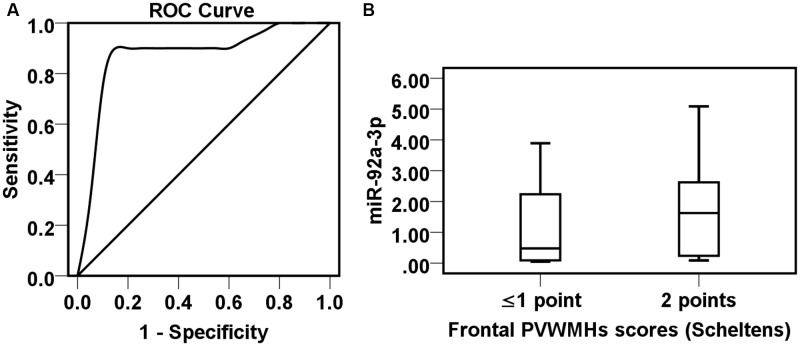
Correlationship among miR-92a-3p, periventricular WMHs (PVWMHs) in frontal caps and early-onset PSD. **(A)** ROC analysis of miR-92a-3p in the early-onset PSD. **(B)** Boxplots comparison of the miR-92a-3p value in patients with different severity of frontal PVWMHs.

We made an ROC analysis of miR-92a-3p and the incidence of early-onset PSD. In a study of 40 samples, ROC analysis revealed that miR-92a-3p could predict early-onset PSD with 90.0% sensitivity and 90.0% specificity, while the optimal cut-off point of miR-92a-3p value is 0.95 [AUC = 0.870 (0.742, 0.998), *p* = 0.000, **Figure 1A**]. Plasma miR-92a-3p concentration above 0.95 at baseline represents a high risk of PSD.

### Plasma miR-92a-3p and the Severity of Frontal PVWMHs

Furthermore, the correlation of miR-92a-3p and the severity of PVWMHs in frontal caps was analyzed. The result demonstrated that the plasma miR-92a-3p content had an upward trend in patients with severe frontal PVWMHs (Scheltens score = 2) compared to non or mild frontal PVMWHs (Scheltens score ≤ 1) (*p* = 0.098, **Figure 1B**).

## Discussion

Through the present study, we found that frontal PVWMH was an independent predictor of early-onset PSD. Some researchers had studied the association between WMHs and stroke or depression. But the results were inconsistent. Kang et al. (2013) found that severe PVWMH was a predictor of poor prognosis (including neurological deficits, activity of daily living, and cognitive ability) after stroke. Shibata et al. (2016) suggested that PVWMH was associated with senile depression. Kawano et al. (2016) concluded that frontal cerebral blood flow was closely related to the severity of depression. Shi et al. (2014) found that frontal lobe lesions were significantly associated with post-stroke depression in a 1-year follow up study. The results of this study were somewhat similar to the findings above about the frontal lobe, PVWMH, stroke, and depression, which suggested that there was a certain correlation among them. There were mainly two hypotheses about the pathogenesis of PSD: biological mechanism hypothesis (Loubinoux et al., 2012) and social psychological hypothesis (Berg et al., 2003). The biological mechanism hypothesis held that neurotransmitters of noradrenergic neurons and serotonergic neurons went through the basal ganglia and thalamus, bypassed the corona and corpus callosum, and reached the frontal lobe via the deep cortex in the brain, which formed the frontal subcortical circuit (FSC). Damage to the circuit could lead to depression. The impairment of the FSC was not only associated with macroangiopathy, but also associated with cerebral small vessel WMLs (Yang et al., 2015). By the hypothesis, it could be suggested that frontal PVWMHs were probably to be associated with PSD by means of damaging the FSC. Patients with frontal PVWMHs were more likely to develop depression at the acute stage of stroke. Frontal PVWMH was an independent predictor for early-onset PSD. However, some studies had drawn different conclusions. Nys et al. (2005) concluded that the severity of WMHs was not significantly associated with PSD. In the evaluation system, the Montgomery depression scale was used to assess depression severity, and the time point of 3 weeks after stroke was selected for diagnosis. This might cause the different conclusions. In another study, Vataja et al. (2001) found that WMHs were not significantly associated with PSD occurring 3–4 months after stroke. In addition to the assessing time point, the Fazekas scale was used to assess the severity of WMHs, which might lead to a different result since the relative simple design of Fazekas scale. In our study, we adopted the Fazekas scale as an assessment framework, and no positive association was obtained either. Tang et al. (2010) suggested that severe DWMH, but not PVWMH, was an independent predictor for PSD. The choice of a time point of 3 months after stroke onset for diagnosis of PSD and the evaluation tool of Fazekas scale, similarly, might be the reason for different conclusions from this study.

We used two visual rating scale “Fazekas scale” and “Scheltens scale” to evaluate WMH in this study. Compared with the volumetric method, the visual rating scale had lower reliability, lower sensitivity and lower objectivity. However, the visual rating scale has its advantage. Firstly, it is simple and easy to use. It does not depend on the software to calculate the volume. It is more widely applicable to clinical practice. Although the volumetric method is more accurate, it is much more complicated and is difficult to operate due to the restrictions of many conditions. Secondly, the visual rating scale was not only qualitative, but also a semi-quantitative assessment. Studies demonstrated that it still has relatively good reliability and validity in the cross-sectional study. Therefore, we adopted the visual rating scale to assess WMHs. Both of the Fazekas and Scheltens scales had relatively good reliability and validity (Scheltens et al., 1998; Kapeller et al., 2003). Fazekas scale was simple and easy to use. It could be applied in most cases, even on poor-quality MRI scans, and was quite suitable for cross-sectional studies of multicenter or large samples (Leys et al., 1990; Kynast et al., 2017; Li et al., 2017). Meanwhile, the scoring system was validated histopathologically (Fazekas et al., 1993). Whereas the Scheltens scale had a greater range than the Fazekas scale and were found to better identify between groups (Gunning-Dixon and Raz, 2000). The Scheltens scale simultaneously counted the number of DWMHs, and was more detailed in the evaluation of PVWMHs compared with Fazekas scale. Considering both size and number of hyperintensities, the summed scores might provide a surface-based volume score, while an analysis of hyperintensities confined to individual regions was possible (Scheltens et al., 1998; Santos et al., 2015; Richter et al., 2017). As a result, we obtained the positive correlation result between frontal PVWMHs and PSD through Scheltens scale, but not Fazekas scale. This was attributed to the refinement of the regional assessing of Scheltens scale in contrast to the simple region setting of Fazekas scale.

In this study, we focus on the relation between WMHs at baseline (at admission) and early-onset PSD (depression in 2 weeks following cerebral ischemia). We supposed that patients with WMHs at baseline were more likely to develop depression in 2 weeks after stroke. It was beneficial to early identify the high-risk early-onset PSD patients at admission. Furthermore, for the confirmation of the diagnosis of ischemic stroke and the early therapy, we performed the MRI as soon as possible in the acute phase of stroke, mostly within 3 days of admission. Moreover, WMH is a chronic ischemic evolution which changes from several months to years. There was almost no difference of the assessment within 2 weeks. Therefore, we choose the analysis of MRI image within 3 days after stroke and the evaluation of depression in 2 weeks. Nevertheless, it would be better to perform MRI at admission and check it again 2 weeks later to further explore the relation between WMH and PSD. But it is limited to the economics and allocations of large equipment.

This study also demonstrated that the NIHSS scores in the PSD group were significantly higher than those in the non-depressed group. Our previous studies have shown that the expression levels of inflammatory mediators such as C-reactive protein (CRP) and interleukin-6 (IL-6) were greatly elevated in stroke patients with severe neurological deficits (Zeng et al., 2013a,b). It might provide the indirect evidence that the neuroinflammatory mechanism was related with PSD. In recent years, many studies revealed that systemic and local neuroinflammation contribute to not only aging, vascular risk factors such as hypertension, diabetes and atherosclerosis, but also WMH, depression, stroke and PSD (Di Napoli et al., 2012; Loubinoux et al., 2012; Popa-Wagner et al., 2014; Sandu et al., 2015; Becker, 2016; Zhang et al., 2016). The increasement of the pro-inflammatory cytokines such as CRP and IL-6 were found in patients with WMH, depression, stroke and PSD (Popa-Wagner et al., 2014; Sandu et al., 2015; Slevin et al., 2015). The release of inflammatory mediators, the infiltration of monocyte into the injured vessel wall and the microglial activation could lead to continuous oligodendrocyte death, consecutive degeneration of myelinated fibers and cause white matter damage (Sandu et al., 2015). In the other hand, the inflammatory injured cascade was trigged once the ischemia event occurred. The microglial in the ischemic region were activated and produced cytokines and chemokines. The exaggerated neuroinflammation could interfere with serotonin metabolism, and reduce both synaptic plasticity and hippocampal neurogenesis. It results in the development of depressive-like behavior after stroke (Popa-Wagner et al., 2014; Sandu et al., 2015). The inflammation might be one of the key mechanisms leading to PSD.

In the present study, we explored the risk factors for frontal PVWMHs. Logistic regression analysis showed that age and number of lacunes were independent risk factors of frontal PVWMHs. Multiple studies had shown that WMHs were closely related to age (Wiszniewska et al., 2000; de Leeuw et al., 2001; Szolnoki, 2007; Tanaka et al., 2009). With the increase of age, the incidence of WMHs increased. The Rotterdam scan study reported that WMHs occurred in 95% of the elder people aged 60–90 years in a sample of 1077 subjects (de Leeuw et al., 2001). WMHs and lacunar infarctions, both closely related to hypertension, belonged to CSVD. The positive correlation between them we found via the study further supported the argument of the same origin of two pathological changes.

In recent years, with the improvement of knowledge of microRNAs and the development of detection technology, researches on microRNAs and stroke continue to emerge. MicroRNAs, which participated in and regulated many biological processes in central nervous system, were related to endothelial dysfunction, apoptosis, cell proliferation, inflammatory response, oxidative stress, angiogenesis, and neurogenesis (Leentjens et al., 2006; Zhang et al., 2010; Sun et al., 2013; Jiang et al., 2014). Moreover, microRNA was stable in the circulating blood and was potentially a new type of biomarker for treatment and diagnosis (Meader et al., 2014). MiR-92a-3p was one of the members of miR-17-92 cluster in microRNAs. The gene sequence was highly conserved and stably existed in circulating blood. MiR-92a-3p participated in many physiological and pathological activities. In our previous study, differential expressions of microRNAs associated with PSD, including miR-92a-3p, were screened out by gene chip (Zhang et al., 2016), which suggested that miR-92a-3p might be a potential biomarker for the diagnosis of PSD. In this study, we made further efforts to evaluate the diagnostic efficacy of miR-92a-3p for early-onset PSD. The ROC analysis indicated that the content of plasma miR-92a-3p could predict early-onset PSD with 90.0% sensitivity and 90.0% specificity. MiR-92a-3p had a high diagnostic efficacy and was expected to be a new molecular biomarker, providing clues for the screening and monitoring of early-onset PSD. We also attempted to explore the possible mechanism of miR-92a-3p. We found that patients with severe frontal PVWMHs (Scheltens score 2) had an increased trend of plasma miR-92a-3p content in contrast to those with mild lesions (Scheltens score ≤ 1), which suggested that periventricular frontal white matter impairment might be one of the pathogenesis of miR-92a-3p promoting the development of early-onset PSD (*p* = 0.098). The significant statistic analysis might be found by increasing the small sample size. Both *in vivo* and *in vitro* experiments demonstrated that miR-92a was abundantly expressed in endothelial cells. Overexpression of miR-92a could damage endothelial cells, while inhibition of the expression of miR-92a could protect the vascular endothelium (Daniel et al., 2014; Loyer et al., 2014). Besides, endothelial-oligodendrocyte trophic coupling played an important role in WMLs. Angiogenesis and myelin regeneration might maintain the stability of the cerebral white matter and promote the repair of white matter impairment through the coupling interaction (Miyamoto et al., 2014). The high expression of miR-92a-3p in patients of early-onset PSD might cause endothelial damage, disruption of blood–brain barrier, and interference of endothelial-oligodendrocyte coupling, resulted in white matter impairment. While the impairment in periventricular frontal white matter would disturb the FSC, then caused dysfunctions of noradrenergic and serotonergic neurons, and eventually involved in the pathophysiology of PSD.

## Conclusion

Our study suggested that patients of acute cerebral infarction with age-related frontal PVWMHs or a high content of plasma miR-92a-3p at baseline were more likely to develop depression at 2 weeks after stroke. The periventricular frontal white matter impairment was possibly to be one of the mechanisms of miR-92a-3p in the pathogenesis of early-onset PSD. MiR-92a-3p might be a new blood biomarker of early-onset PSD. New ideas might be provided for the therapy of PSD via the regulation of the expression of miR-92a-3p. But there are several limitations in the current study. Firstly, the sample size of the study was small. The results should be further confirmed in a large cohort of stroke patients. Secondly, MRI image and the evaluation of depression were not carried out simultaneously. MRI was performed within 3 days following cerebral ischemia, and the depression was evaluated in 2 weeks after stroke. For better explore the relation between WMH and PSD, we might as well have the second check image in 2 weeks. Thirdly, the assessment methods of WMH in the study were visual rating scale. The volumetric methods might be suggested because of greater precision.

## Ethics Statement

This study was carried out in accordance with the recommendations of ‘Study on related factors and biomarkers of post stroke depression, Ethics Committee of Ruijin Hospital, Shanghai Jiaotong University School of Medicine’ with written informed consent from all subjects. All subjects gave written informed consent in accordance with the Declaration of Helsinki. The protocol was approved by the ‘Ethics Committee of Ruijin Hospital, Shanghai Jiaotong University School of Medicine.’

## Author Contributions

LZ involved in all aspects of the study, supervised tissue assay analysis. She participated in the study design, data analysis and the revision of the article. JH drafted the enclosed manuscript, performed the evaluation of MRI image and the analysis of data. YZ performed the collection of blood samples and clinical evaluation in stroke patients. WL, HL, and XT performed experiments on patient’s samples and the revision of the article. FM and GY participated in the design of the present study and revision of the article.

## Conflict of Interest Statement

The authors declare that the research was conducted in the absence of any commercial or financial relationships that could be construed as a potential conflict of interest.
